# Effectors of Epidermal Growth Factor Receptor Pathway: The Genetic Profiling of *KRAS*, *BRAF*, *PIK3CA*, *NRAS* Mutations in Colorectal Cancer Characteristics and Personalized Medicine

**DOI:** 10.1371/journal.pone.0081628

**Published:** 2013-12-10

**Authors:** Yinchen Shen, Jianfei Wang, Xiaohong Han, Hongying Yang, Shuai Wang, Dongmei Lin, Yuankai Shi

**Affiliations:** 1 Department of Medical Oncology, Cancer Institute/Hospital, Peking Union Medical College and Chinese Academy of Medical Sciences, Beijing, China; 2 Beijing Key Laboratory of Clinical Study on Anticancer Molecular Targeted Drugs, Beijing, China; 3 Department of Pathology, Cancer Institute/Hospital, Peking Union Medical College and Chinese Academy of Medical Sciences, Beijing, China; The Chinese University of Hong Kong, Hong Kong

## Abstract

Mutations in *KRAS* oncogene are recognized biomarkers that predict lack of response to anti- epidermal growth factor receptor (EGFR) antibody therapies. However, some patients with *KRAS* wild-type tumors still do not respond, so other downstream mutations in *BRAF, PIK3CA* and *NRAS* should be investigated. Herein we used direct sequencing to analyze mutation status for 676 patients in *KRAS* (codons 12, 13 and 61), *BRAF* (exon 11 and exon 15), *PIK3CA* (exon 9 and exon 20) and *NRAS* (codons12, 13 and 61). Clinicopathological characteristics associations were analyzed together with overall survival (OS) of metastatic colorectal cancer patients (mCRC). We found 35.9% (242/674) tumors harbored a *KRAS* mutation, 6.96% (47/675) harbored a *BRAF* mutation, 9.9% (62/625) harbored a *PIK3CA* mutation and 4.19% (26/621) harbored a *NRAS* mutation. *KRAS* mutation coexisted with *BRAF*, *PIK3CA* and *NRAS* mutation, *PIK3CA* exon9 mutation appeared more frequently in *KRAS* mutant tumors (P = 0.027) while *NRAS* mutation almost existed in *KRAS* wild-types (P<0.001). Female patients and older group harbored a higher *KRAS* mutation (P = 0.018 and P = 0.031, respectively); *BRAF* (V600E) mutation showed a higher frequency in colon cancer and poor differentiation tumors (P = 0.020 and P = 0.030, respectively); proximal tumors appeared a higher *PIK3CA* mutation (P<0.001) and distant metastatic tumors shared a higher *NRAS* mutation (P = 0.010). However, in this study no significant result was found between OS and gene mutation in mCRC group. To our knowledge, the first large-scale retrospective study on comprehensive genetic profile which associated with anti-EGFR MoAbs treatment selection in East Asian CRC population, appeared a specific genotype distribution picture, and the results provided a better understanding between clinicopathological characteristics and gene mutations in CRC patients.

## Introduction

Colorectal cancer (CRC) still causes majority of mortality in the world [1]. In mCRC tumors, exceedingly poor prognosis was observed. Fortunately, the rapid development in biological agents appears a promising future in treatment. Cetuximab or panitumumab, the monoclonal antibody (MoAb) targeted on epidermal growth factor receptor (EGFR), has been implemented in clinical practice, and emerged as an effective single agent or chemotherapy adjuvant approach for mCRC treatment [2]. These MoAbs blocks the downstream intracellular signaling of EGFR, which includes two main signaling pathways, RAS-RAF-MAPK axis, which mainly involved in cell proliferation, and the phosphatidylinositol 3-kinase (PI3K)-PTEN-AKT, key mediators of survival, and motility-invasion [3].

Although previous clinical trials have indicated that patients who carry *KRAS* mutations in codons 12 and 13 are non-responsive to the EGFR-targeted therapy [4], [5], [6], [7], and the wild-type status seems a response condition, some wild-type patients still fail to respond to anti-EGFR monoclonal antibody therapy [8], and the mechanism remains unclear. It is possible that mutations in the downstream effectors of the EGFR signaling pathway, such as *BRAF*, *PIK3CA* and *NRAS*, may induce a negative effect on the response in anti-EGFR targeted treatment [9], [10], [11].

To date, genetic profiling of individual tumors affect the selection of therapy and treatment response have been proven in clinical practice, however, major data about the frequency of oncogenes mutations were presented in Western populations and few data are available for the Chinese. Since gene mutation status alters with ethnic differences [12], we design this study to investigate the ethnicity-specific role of mutations in development and progression of CRC. *KRAS*, *BRAF*, *PIK3CA* and *NRAS* mutations in primary tumors from Chinese CRC patients were detected and their potential correlations with clinicopathological factors were analyzed. Furthermore, we collected the survival data of mCRC subgroup patients, in order to obtain an appropriate insight between gene mutation and survival status. We intended that these data could benefit the design of future clinical trials and individualized therapy in CRC patients.

## Materials and Methods

### Patients

We investigated 676 consecutive patients who underwent surgery for colorectal cancer at the Cancer Institute/Hospital of the Chinese Academy of Medical Sciences (Beijing, China) between August 2010 and December 2011, all the patients were carried out primary resection in our hospital, and no patient had received chemotherapy before surgery. Each patient was contacted to provide available formalin-fixed, paraffin-embedded (FFPE) CRC tissues. Written informed consent was obtained from individual patients, and the experimental protocol was approved by the Institutional Review Board (IRB) in Cancer Institute/Hospital of Chinese Academy of Medical Sciences and Peking Union Medical College. CRC diagnosis was confirmed by hematoxylin and eosin (HE) staining and histological analysis. Overall survival was defined as the period from the start of diagnosed CRC until death from any cause or last follow-up. The patients’ demographic and clinicopathological data are presented in Table 1.

**Table 1:** Characteristics of 676 CRC patients and association of gene mutations with clinicopathological parameters.

Characteristics	Number (%)	KRAS		BRAF		PI3KCA		NRAS	
		Mutations (%)	*P*	Mutations (%)	*P*	Mutations (%)	*P*	Mutations (%)	*P*
Sex									
Male	407 (60.2)	131 (32.3)	0.02	28 (6.9)	0.93	34 (9.0)	0.35	16 (4.3)	0.93
Female	269 (39.8)	111 (41.3)		19 (7.1)		28 (11.3)		10 (4.0)	
Age									
≤60	342 (50.6)	109 (32.0)	0.03	23 (6.7)	0.81	25 (8.0)	0.11	15 (4.9)	0.38
>60	334 (49.4)	133 (39.9)		24 (7.2)		37 (11.9)		11 (3.5)	
Mean	60±11								
Range	23–86								
Primary tumor site									
Rectum	391 (57.8)	138 (35.4)	0.74	27 (6.9)	0.96	22 (6.1)	<0.001	19 (5.4)	0.09
Colon^†^	285 (42.2)	104 (36.6)		20 (7.0)		40 (15.1)		7 (2.6)	
Tumor location*									
Proximal	133 (19.7)	52 (39.1)	0.38	9 (6.8)	0.92	25 (19.8)	<0.001	4 (3.1)	0.51
Distal	542 (80.2)	189 (35.0)		38 (7.0)		37 (7.4)		22 (4.5)	
Missing data	1 (0.1)								
Tumor differentiation									
Well	31 (4.6)	17 (54.8)	0.06	1 (3.2)	0.18	4 (14.3)	0.21	2 (6.9)	0.07
Moderate	556 (82.2)	197 (35.6)		36 (6.5)		46 (9.0)		17 (3.4)	
Poor	87 (12.9)	27 (31.0)		10 (11.5)		12 (14.5)		7 (8.4)	
Missing data	2 (0.3)								
Tumor stage↑									
I	92 (13.6)	33 (35.9)	0.56	5 (5.4)	0.61	8 (9.9)	0.29	4 (4.9)	0.03
II	238 (35.2)	86 (36.1)		17 (7.1)		20 (9.0)		8 (3.6)	
III	288 (42.6)	107 (37.4)		19 (6.6)		25 (9.3)		7 (2.6)	
IV	55 (8.1)	15 (27.3)		6 (10.9)		9 (18.0)		6 (12.2)	
Missing data	3 (0.5)								
Depth of invasion↑									
T1	18 (2.7)	5 (27.8)	0.21	1 (5.6)	0.57	0 (0.0)	0.34	1 (6.3)	0.69
T2	105 (15.4)	37 (35.6)		4 (3.8)		9 (9.7)		4 (4.2)	
T3	521 (77.1)	184 (35.4)		40 (7.7)		48 (9.9)		20 (4.2)	
T4	30 (4.5)	16 (53.3)		2 (6.7)		5 (17.2)		0 (0.0)	
Missing data	2 (0.3)								
Lymph node↑									
N0	342 (50.8)	123 (36.0)	0.88	23 (6.7)	0.48	30 (9.5)	0.75	12 (3.8)	0.89
N1	190 (28.0)	66 (34.9)		11 (5.8)		16 (9.4)		8 (4.7)	
N2	142 (20.9)	53 (37.6)		13 (9.2)		16 (11.7)		5 (3.8)	
Missing data	3 (0.3)								
Distant metastasis									
Yes	55 (8.1)	15 (27.3)	0.16	6 (10.9)	0.26	9 (18.0)	0.08	6 (12.2)	0.01
No	619 (91.6)	227 (36.8)		41 (6.6)		53 (9.2)		19 (3.3)	
Missing data	2 (0.3)								

Colon *: Colon is defined as right colon, transverse colon, left colon, *sig*moid colon, rectosigmoid transition zone.

Tumor location *: Proximal tumor is defined as right colon and transverse colon; distal tumor is defined as left colon, *sig*moid colon, rectosigmoid transition zone and rectum.

†: Seventh edition of the AJCC/UICC TNM staging systems.

### DNA Extraction and Mutation Analysis

Before the extraction of genomic DNA, all CRC samples were identified by two pathologists in order to ensure the representative malignant cells exist in each sample, the tissue blocks were cut into 5 µm sections, then microdissection was performed to guarantee each tissue sample tested contained >80% cancer cells. DNA was extracted by the QIAamp DNA Blood Mini Kit (Qiagen, Hilden, Germany) according to the manufacturer’s instructions and stored at −80°C until use.

We detected the mutation hotspots in *KRAS* (codons 12 and 13), *BRAF* (exon15), *PIK3CA* (exon 9 and exon 20) and *NRAS* (codon 61), where the most mutations occur in these genes [13], [14], besides, rare types of mutants for *KRAS* (codon 61), *BRAF* (exon 11) and *NRAS* (codons 12 and 13) were also included. The program for the PCR amplification in *KRAS*, *BRAF*, *NRAS* and *PIK3CA* exon20 was as follows: 1 min of initial denaturation at 95°C, 35 cycles of amplification consisting of 30 s at 94°C, 40 s at 57°C, and 30 s at 72°C, with a final additional elongation at 72°C for 7 min. *PIK3CA* exon 9 amplification was carried out with a touchdown PCR program: 94°C (2 min); 3 cycles of 94°C (30 sec), 64°C (30 sec), 70°C (30 sec); 3 cycles of 94°C (30 sec), 61°C (30 sec), 70°C (30 sec); 3 cycles of 94°C (30 sec), 58°C (30 sec), 70°C (30 sec); 32 cycles of 94°C for (30 sec), 57°C (30 sec), 70°C (40 sec); 1 cycle of 70°C (5 min). When performing the PCR, a non-template control was included in each batch. After PCR reaction, the products were purified and subjected to direct sequencing (ABI 3500×L Genetic Analyzer; Applied Biosystems, Carlsbad, CA, USA).

### Statistical Analysis

Statistical analysis was carried out by the SPSS 17.0 statistical software (SPSS, Inc., Chicago, IL, USA). The Chi-square (χ^2^) test was used to compare the proportion of gene mutations among groups with different clinicopathologic factors. Multiple logistic regression analysis was done to investigate the effects of covariates on gene mutations, using a backward stepwise (likelihood ratio) method with odds ratio (OR) calculated, and variables which showed statistically significant association with gene mutations were subjected to final regression analysis. Survival analysis was done with the Kaplan-Meier survival function with the method of log-rank test. The two-sided significance level was set at *P*<0.05.

## Results

### *KRAS* Mutation

**Figure 1:**
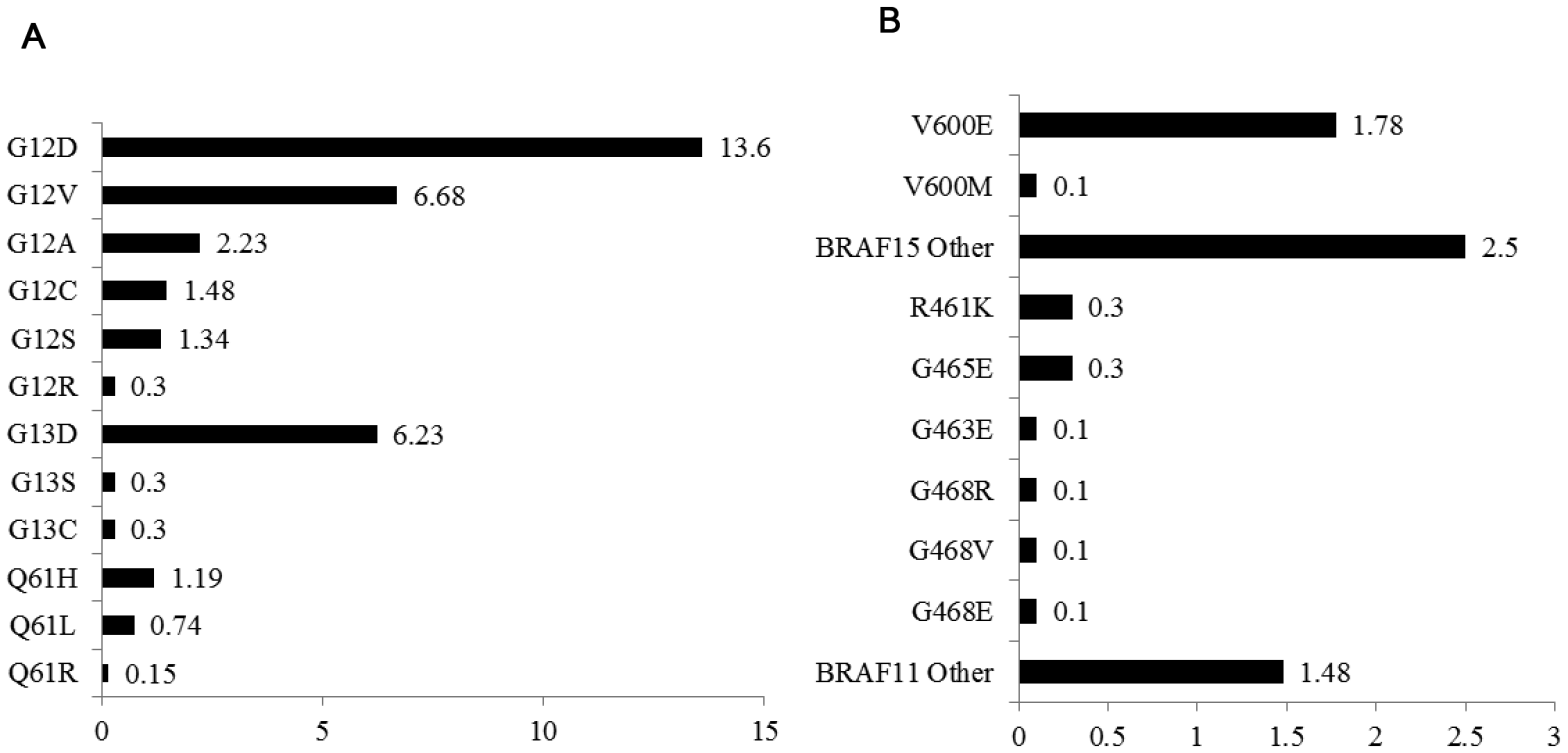
Frequency of the various KRAS and BRAFmutations. Panel A: KRAS mutations (codons12 & 13: n = 674; codon61: n = 672). Panel B: BRAF mutations (exon11: n = 676; exon15: n = 675). The data are presented as percentages (number of total samples).

*KRAS* mutation status could not be assigned to 2 of 676 (0.30%) samples, 35.9% (242/674) harbored a *KRAS* mutation, 25.7% (173/674) in codon12, 6.8% (46/674) in codon13, and 2.1% (14/672) in codon61. Moreover, one patient harbored a double *KRAS* mutation in both codon12 and 13 (GGT>GTT, GGC>AGC). The corresponding order for *KRAS* codon12 mutation frequency was G12D, G12V, G12A, G12C, G12S and G12R; in *KRAS* codon13, the most frequent mutation was G13D, followed by G13C and G13S. The major mutation subtype in codon61 was Q61H, and Q61L, Q61R were also found in this study (Figure 1). *KRAS* mutation appeared more frequently in female than male (41.3% vs 32.3%, P = 0.02), and patients older than 60 years also showed a higher rate of *KRAS* mutation (39.9% vs 32.0%, P = 0.03). We did not find other significant associations between *KRAS* mutation and patients’ clinicopathological characteristics (Table 1).

### *BRAF* Mutation

The status of *BRAF* mutation was detected in 99.8% (675/676) samples, 6.96% (47/675) harbored a *BRAF* mutation, 4.4% (30/675) in exon15 and 2.5% (17/676) in exon11. The V600E mutation in exon15 was the most frequent subtype (1.8%,12/675), and followed by V600M mutation and other types. In exon11, the mutations distributed widely, R461K and G465E were relatively more common in these mutations (Figure 1). *BRAF* and *KRAS* mutations were not mutually exclusive, 4.55% (11/242) of *KRAS* mutant tumors harbored a *BRAF* mutation (of which 7/242[2.89%] exon15 and 4/242[1.66%] exon 11 mutations). However, *BRAF* (V600E) only existed in *KRAS* wild types (0.0% vs 2.78% [12/431], P = 0.005). In this group, V600E mutation showed a strong association with primary tumor site, tumor in colon appeared more frequently to harbor a V600E mutation (9/285[3.2%] in colon vs 3/390[0.8%] in rectum; P = 0.020), besides, with the tumor differentiation getting worse, a higher V600E mutation rate emerged (5/87[5.7%] in poor differentiation vs 7/555[1.3%] in moderate differentiation; P = 0.030). No other significant association was found between *BRAF* mutation and patients’ characteristics (Table 1).

### *PIK3CA* Mutation

**Figure 2:**
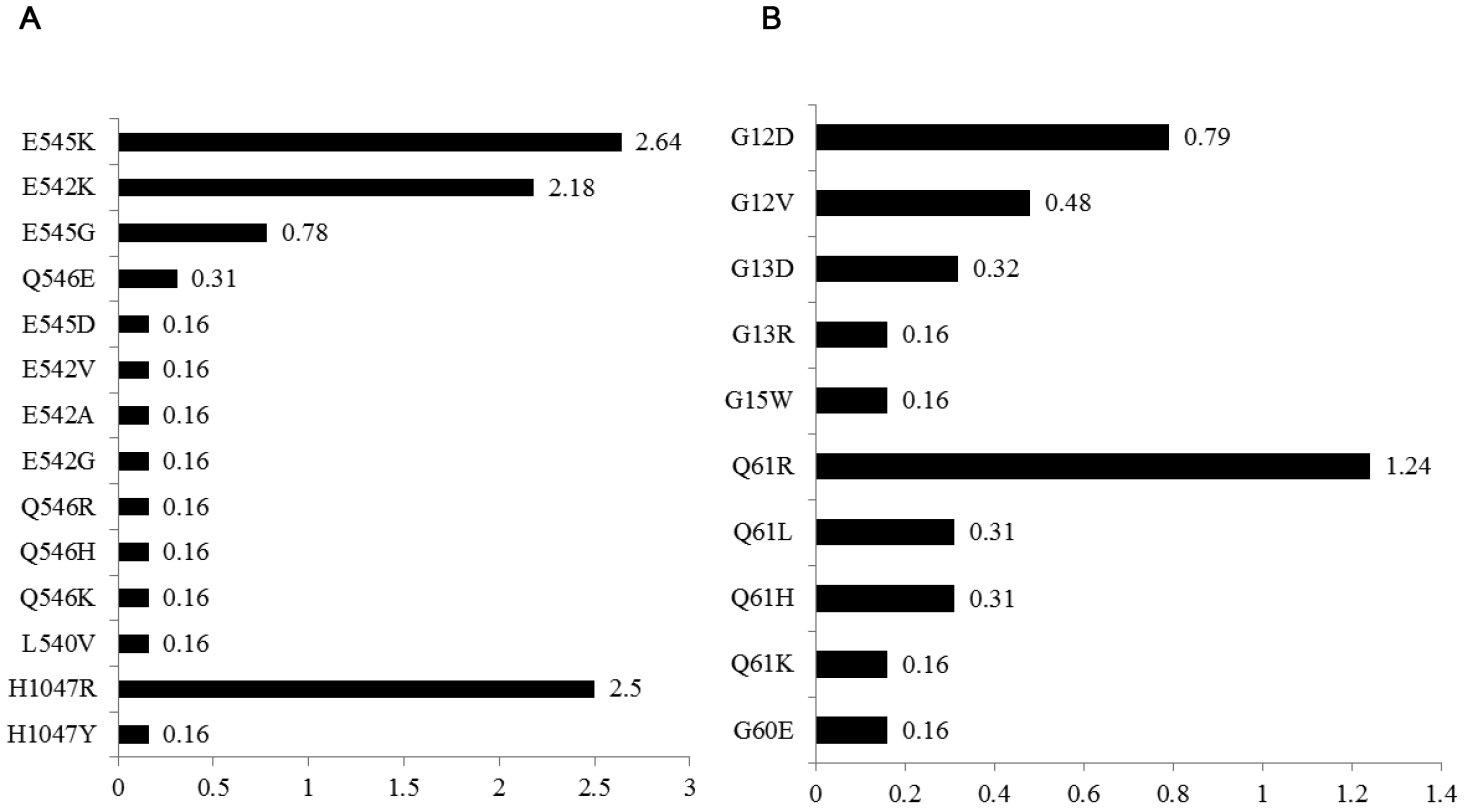
Frequency of the various PIK3CA and NRAS mutations. Panel A: PIK3CA mutations (exon9: n = 643; exon20: n = 636). Panel B: NRAS mutations (codons12 & 13: n = 630; codon61: n = 643). The data are presented as percentages (number of total samples).

*PIK3CA* mutation status could not be assigned to 7.54% (51/676) samples, 9.9% (62/625) harbored a *PIK3CA* mutation, 7.0% (45/643) in exon9 and 2.67% (17/636) in exon20. The E545K in exon9 appeared more frequently than any other mutation subtype, followed by E542K, E545G, Q546E and others. By contrast, nearly all mutations in exon20 was H1047R, only one sample was H1047Y mutation, the spectrum of these mutations was showed in Figure 2. We also detected one sample harbored a double mutation in exon9 (L540V and Q546E), besides, this sample also had a *KRAS* mutation (G13D). There was a strong significant association between *PIK3CA* exon9 and *KRAS* mutations (23/230[10.0%] in *KRAS* mutant vs 22/412[5.3%] in *KRAS* wild types, P = 0.027), whereas this association was not found in *PIK3CA* exon20 with *KRAS* mutation (P = 0.673). *BRAF* and *PIK3CA* mutations were not mutually exclusive, 10% (4/40) of *BRAF* mutation coexists with *PIK3CA* mutation (of which 3/40[7.5%] exon 9 and 1/40[2.5%] exon20). For the clinicopathological characteristics analysis, patients with tumor located in rectum had a significantly lower *PIK3CA* mutation rate than other sites in colon and rectosigmoid transition zone (6.1% vs 15.1%, P<0.001) and proximal tumors appeared a higher *PIK3CA* mutation rate (19.8% vs 7.4%, P<0.001). No other significant association was found in this analysis (Table 1).

### *NRAS* Mutation

We detected *NRAS* mutation in 92.0% (621/676) samples, and 4.19% (26/621) harbored a *NRAS* mutation. Although *NRAS* is closely to *KRAS* which also included in Ras gene [13], unlike *KRAS*, most *NRAS* mutation occurred in codon61 (2.02%, 13/643), rather than in codon12 or 13 (1.75%, 11/630). The most frequently mutation subtype in codon61 was Q61R, and G12D in codon12/13 (Figure 2). Besides, one G15W and one G60E mutation were also detected in these samples. Moreover, we still found that *NRAS* mutation appeared a strong significant association with *KRAS* wild types (1/227[0.44%] in *KRAS* mutant vs 25/394[6.3%] in *KRAS* wild types, P<0.001). Interestingly, *NRAS* codon61 mutation only harbored in *KRAS* wild types (0.0% vs 3.2% [13/410], P = 0.006), whereas *NRAS* codon12 and 13 did not share this association (P = 0.063). Only one sample harbored a *BRAF* mutation (V600E) with a *NRAS* mutation (G15W), and 6.78% (4/59) *PIK3CA* mutation harbored a *NRAS* mutation (of which 2/59[3.39%] in codons 12 and 13, 2/61[3.28%] in codon61). Furthermore, *NRAS* mutation occurred more frequently in distant metastasis tumors (12.2% vs 3.3%, P = 0.010), and different tumor stage showed a different *NRAS* mutation rate (P = 0.030). (Table 1).

In the multivariate logistic regression analysis, we selected sex, age, primary tumor site, tumor differentiation, tumor stage and distant metastasis as covariates, and *KRAS* mutants appeared more frequently in patients older than 60 (P = 0.023), as well in female patients (P = 0.016). *BRAF* mutations did not show any significant association with characteristics (data not shown), while *BRAF* (V600E) mutants shared significant association with tumor differentiation (P = 0.016). As for *PIK3CA* mutations, colon cancer appeared a higher mutation rate than rectum cancer (P<0.001), however, *NRAS* mutations showed more frequently in rectum cancer (P = 0.031), although no significant association was found in univariate analysis (P = 0.09). Moreover, a strong significant association still existed between *NRAS* mutants and distant metastasis in the multivariate analysis (Table 2).

**Table 2:** Multivariate logistic regression in CRC patients between gene mutations and clinicopathological characteristics.

Characteristics	KRAS			BRAF (V600E)	
	Adjusted odds ratio (95% CI)	LRT p value		Adjusted odds ratio (95% CI)	LRT p value
Sex	0.671(0.485–0.928)		0.016	0.627(0.197–1.994)	0.429
Age	1.450(1.052–1.999)		0.023	1.241(0.381–4.040)	0.720
Primary tumor site	1.066(0.768–1.480)		0.702	3.587(0.947–13.588)	0.060
Tumor differentiation	0.675(0.453–1.006)		0.053	4.101(1.298–12.957)	0.016
Tumor stage	1.098(0.864–1.396)		0.443	1.310(0.601–2.855)	0.496
Distant metastasis	0.681(0.362–1.280)		0.232	0.637(0.069–5.891)	0.691
**Characteristics**	**PIK3CA**			**NRAS**	
	**Adjusted odds ratio (95% CI)**	**LRT p value**		**Adjusted odds ratio (95% CI)**	**LRT p value**
Sex	0.766(0.447–1.315)	0.334		1.394(0.587–3.311)	0.452
Age	1.491(0.869–2.559)	0.147		0.700(0.305–1.609)	0.401
Primary tumor site	2.773(1.604–4.792)	<0.001		0.348(0.134–0.907)	0.031
Tumor differentiation	1.025(0.534–1.970)	0.940		1.587(0.609–4.138)	0.345
Tumor stage	0.939(0.616–1.432)	0.771		0.708(0.384–1.305)	0.268
Distant metastasis	1.802(0.814–3.989)	0.146		4.930(1.817–13.375)	0.002

LRT: likelihood ratio test; 95% CI: 95% confidence interval.

### Analysis of Gene Mutation in mCRC Patients

Fifty-five of 676 patients were confirmed as mCRC, and all these 55 samples were collected before chemotherapy. We further investigated the mutants distribution and clinicopathological characteristics association in this group. 27.3% (15/55) harbored a *KRAS* mutation, of which 93.3% (14/15) in codon12 and 6.7% (1/15) in codon13, respectively. The *BRAF* mutation rate was 10.9% (6/55), 66.7% (4/6) in exon15 and 33.3% (2/6) in exon11. *PIK3CA* mutation was detected in 18.0% (9/50) tumors, 66.7% (6/9) was detected in exon9 and 33.3% (3/9) in exon20. 12.24% (6/49) tumors were detected as *NRAS* mutants, of which 50% (3/6) in codons12 and 13, 33.3% (2/6) in codon61, besides, one sample harbored a G15W mutation. Statistical analysis indicated that *KRAS* mutation was significantly higher in the deeper invasion stage (5/7[71.4%] in T4 vs 10/48[20.8%] in T3; OR 9.500, 95% CI 1.599–56.426; P = 0.013), and tumor with poor differentiation showed a higher *NRAS* mutation rate than moderate differentiation (5/19[26.3%] vs 1/30[3.3%]; OR 10.357, 95% CI 1.103–97.266; P = 0.027). We did not find any other significant association between gene mutation (included subgroup analysis) and clinicopathological characteristics (data not shown).

### Overall Survival Analysis in mCRC Patients

Overall survival of patients in this subgroup was analyzed with the Kaplan-Meier method, in this relative small subgroup (n = 55), survival information was collected successfully in only 45 patients, of whom 37 had received chemotherapy after surgery, either with infusional fluorouracil, leucovorin and irinotecan (FOLFIRI) or infusional fluorouracil, leucovorin, and oxaliplatin (FOLFOX4). However, the relative small sample size did not present any significant result between gene mutation and OS, including gene subsets analysis (data not shown).

## Discussion

During the past decades, large amounts of research data emerged from molecular basis [15], drug investigation and usage [3], genetic profiling effects [16] studies have led to the thriving research on identification of multiple molecular subsets and targeted therapy in colorectal cancer. Following the discovery that mutant *KRAS* tumors were resistant to anti-EGFR antibodies, patients with metastatic colorectal cancer are now recommended to detect the *KRAS* codons12 and 13 mutation status before MoAbs therapy [8], [17], [18]. However, even in *KRAS* wild-type tumors, up to 65% patients were still resistant to anti-EGFR monoclonal antibodies [8]. Besides, although the detection of *KRAS* mutation status before MoAbs therapy is widely accepted, there is little agreement on its predicted and prognostic role, for published studies provided different results in the relationship between *KRAS* mutation and clinical outcomes in CRC, and the main effectors in downstream signaling pathway of *KRAS*, such as *BRAF*, *PIK3CA* and *NRAS* were already studied in many clinical trials, which showed the capability to present as potential predictive or prognostic biomarkers [14], [19].

To our knowledge, this study investigated the first time gene mutation type distribution in Chinese CRC population, and involved not only *KRAS*, but also *BRAF*, *PIK3CA*, *NRAS* together for comprehensive analysis between gene mutation and clinicopathological characteristics, in addition, the overall survival of metastatic colorectal cancer. Previous studies usually focused on *KRAS*, *BRAF*, *PIK3CA* [20], [21], [22], but not include *NRAS* mutation, or study sample size was too small to draw confirmed conclusions [20]. Many studies could not collect enough appropriate samples to describe a relative complete outline for Chinese CRC patients in genetic profile, and our investigation aimed to present the key mediated gene mutation of CRC, to some extent, representing the East Asian population.

*KRAS* gene encodes a small G protein which acts as a key transducer in EGFR pathway, mutations in *KRAS* gene lead to constitutive signaling through the EGFR pathway and active downstream MAPK and *PIK3CA* dependent pathways [18], [23]. Previous studies have analyzed *KRAS* mutation distribution from western population, which indicated that G12D was the most frequent mutation subtype in codon12, followed by G12V, G12C, G12S, G12A and G12R [24]. However, in present study, the corresponding order for *KRAS* codon12 mutation frequency was G12D, G12V, G12A, G12C, G12S and G12R. As for codon13, the difference remained in subtype distribution (G13/D/C/R in western population vs G13/D/C/S in this study). In addition to gaining more information and expanding the recognition of the *KRAS* mutation, the sample size of our series allowed us to investigated the rare codon61 mutation, since mutant tumors with *KRAS* codon61 led to significantly lower response rate than wild types (0.0% vs 35.7%, P = 0.0055) [14], while the mutation incidence (2.1%) was even higher than some codon12 and 13 mutations, we then suggested that codon61 detection should be taken into consideration during clinical practice. This study showed a 35.9% *KRAS* mutation rate, which was similar to previous studies [4], [9], [14], [22], and patients older than 60 appeared more frequently to harbor a *KRAS* mutation. Meanwhile, in mCRC patients, *KRAS* mutation was significantly higher in the deeper invasion stage (OR 9.500, 95% CI 1.599–56.426; P = 0.013), which was consistent with Li HT and colleagues [25] reported that *KRAS* mutation had a strong association with Dukes’ staging, with the highest mutation rate in Dukes’ D staging tumors. Our molecular data provided an evaluation of possibility for disease progression. Then the patients with rapid distant metastasis seemed more likely to be initial resistant to anti-EGFR MoAbs, because *KRAS* mutation maintained throughout the CRC development, progression and metastasis, with a high (95%) concordance presenting at the primary and related metastatic sites [26]–[27].

*BRAF* gene is a member of the RAF gene family, which encodes a serine-threonine protein kinase, acts as a downstream effector of activated KRAS. Previous studies reported that *KRAS* and *BRAF* mutations were mutually exclusive in mCRC, and *BRAF* mutation occurring in approximately 5%–10% tumors [14], [28], [29]. However, in this study, we found that *KRAS* and *BRAF* mutations were not mutually exclusive, 4.55% (11/242) of *KRAS* mutant tumors harbored a *BRAF* mutation, our data were supported by Mao C and colleagues [20], although they gained an extremely high *BRAF* mutation incidence (25.4%,15/59), for reported studies of *BRAF* mutations usually presented a higher mutation frequency in western population (8.5%–13.9%) [29]–[30] than the Chinese (1.1%–7.0%) [25], [31]. In our study, BRAF mutation (6.96%) was consistent with previous results, and the lower BRAF mutation frequency may attribute to the patients population studied. However, difference in mutation frequency also indicates that geographical and ethnical variations play a role in gene mutation distribution. In the reported studies, most of the data were collected from only *BRAF* V600E mutation type, other types such as V600M were not included [14], [29], while we also confirmed this point for *BRAF* (V600E) only existed in *KRAS* wild types (0.0% vs 2.78% [12/431], P = 0.005). However, *BRAF* mutation associated with poor clinical outcomes were proven in several studies [10], [14], [29], herein we reported a *BRAF* mutation rate for 4.4% (30/675) in exon15 and 2.5% (17/676) in exon11, which were both higher than *KRAS* codon61 (2.1%) mutation. As De Roock W and colleagues [14] recommended *BRAF* should be tested subsequently after *KRAS*, we supposed both *BRAF* exon15and 11 need to be taken into consideration, in order to select better suitable subgroup patients. In addition, V600E mutation was significantly higher in colon cancer than rectum cancer (OR 4.035, 95% CI 1.062–15.330; P = 0.041) and poor differentiation tumor harbored a higher V600E mutation (P = 0.030). These data indicated that colorectal cancer treatment should be regarded from a deeper extent, for colon and rectum cancer required different therapy in different stage.

We confirmed the association between *KRAS* and *PIK3CA* mutations in CRC, which was comparable with previous studies [14], [20], [25], [32], and only exon9 (not included exon20) shared a strong association with *KRAS* mutation [14]. This was consistent with the findings that the gain of function by exon9 mutations (the helical domain) was highly dependent on RAS-GTP binding, especially in E542K and E545K, while exon20 mutations (the kinase domain) active was likely in the absence of RAS-GTP binding [33]. The *PIK3CA* mutation frequency varies between 13.6%–18.0% in western population [34]–[35], while we reported a relative low mutation frequency (9.9%). Studied population may lead to this difference mainly, for other studies which based on Chinese population also showed lower mutation frequency (4.9%–8.2%) [20], [36]. In the logistic regression analysis, *PIK3CA* mutation appeared more frequently in colon cancer than rectum at the same time, which was supported by a recent study [37]. Previous studies indicated that *PIK3CA* mutation existence implied negative prognosis, either a shorter median progression-free survival (PFS) [38], or a shorter median OS [39]–[40]. However, since the *PIK3CA* mutation effect seemed to be considered together, the separate effect of each subtype appeared unclear, for several studies had showed that exon9 and exon20 mutation led to different results [14], [41]. The large European consortium study indicated than only exon20 mutation was associated with worse clinical outcome [14], and was supported by other research data [16], [42]. But because exon20 mutation was relatively low compared with exon9 (2.96% vs 9.96%) [14], and in our study (2.67% vs 7.0%). The reported data should be regarded as clinical related hypothesis and required confirmation, based on further genetic profiling and clinical trials investigation.

The RAS gene (KRAS, NRAS, HRAS) encodes a series of GTP/GDP related switches that convey extracellular signals, resulting in regulating growth and survival of cells [43]. As one of the RAS family, *NRAS* shared close relations with *KRAS* [13], while unlike *KRAS* mutation occupies such a large percentage in colorectal cancer, *NRAS* mutations were rare. Irahara N and colleagues [44] reported a 2.2% (5/225) mutation incidence, and 2.64% (17/644) mutation rate in another study [14], while we detected 4.19% (26/621) tumors harbored a *NRAS* mutation. The higher NRAS mutation incidence presented a specific characteristic for Chinese population. As rare data was reported in Chinese patients for NRAS mutation status, our study may provide some original contribution. However, future investigations are needed to draw a better picture in this area. *NRAS* mutations were not mutually exclusive with *BRAF* and *PIK3CA* mutation, although another study did not share this [44]. *NRAS* mutation coexisted with *KRAS* wild-type (P<0.001), of note, codon61 mutation only appeared in *KRAS* wild-type tumors (P = 0.006), and codon12 and 13 had a significantly higher mutation rate in distant metastatic tumors (P = 0.016). These data can partially help explain the anti-EGFR MoAbs resistance in *KRAS* wild-type patients, as *NRAS* mutations were significantly associated with lower disease control rate and response rate to MoAbs [14], [45], and we recommended *NRAS* mutation detection should be taken into consideration before MoAbs treatment, especially in *KRAS* wild-type tumors. However, considering the low mutation incidence, the magnitude of *NRAS* mutation effect was still confused, larger sample size or preselected patients investigation seemed essential in future design.

There were several limitations in this retrospective study, including the relatively small number (n = 45) of patients in the survival analysis, then the limited information could not support confirmed conclusions in present study. Additionally, other potentially negative factors such as loss of expression of phosphatase and tensin homologue (PTEN) should be involved, thus essential effects of these biomarkers in clinical practice stayed further validation. Moreover, as epigenetic status or microsatellite instability (MSI) plays a significant role in CRC tumors, these features should be involved into analysis. Besides, gene expression in the key effectors, different tumor locations may provide information for better understanding in CRC, either in carcinogenesis or tumor progression and these should be taken into consideration in future studies. As high throughput detecting method has been implemented in screening gene variants or sequencing, different types of gene alternations have been investigated comprehensively in colorectal cancer [46], these studies have provided potential genes which need further investigations.

In a recent randomised trial [47], patients were preselected for only *KRAS* codon12, 13 and 61 wild-type tumors, however, therapy with panitumumab to irinotecan did not improve the overall survival compared with irinotecan alone, then refinement of molecular selection was required considering patients’ welfare. Another multicenter randomised placebo-controlled trail tested a novel multikinase inhibitor (Regorafenib) [48], although the study obtained a significant result in prolonging median OS (6.4 vs 5.0 months, hazard ratio 0·77; 95% CI 0·64–0·94; one-sided p = 0·0052), in view of the small incremental survival benefit, potentially exposed to toxic effects and heavy economic burden, the new agent seemed not to be a cost-effective option, while selecting the subset of patients who would really benefit from Regorafenib based on the identification of biomarkers was a high priority. We have already known that genotype subgroup would lead to different clinical outcomes in mCRC MoAbs treatment [14], [49], [50], all the data indicated that more precise classification of genetic profile should be implemented to enhance the clinical targeted therapy, then our study here was in order to bring us a step closer to personalized medicine.

In conclusion, this study presented a clear genotype distribution picture scroll in East Asian CRC population, involving potential molecular predictors *KRAS*, *BRAF*, *PIK3CA*, *NRAS*, which showed a specific characteristic. However, prospective randomised trials are needed to provide proposals and validate conclusions. More comprehensive genomic analysis and molecular classification should be performed, to recognize the genetic profile better and to improve the clinical choice smarter.
